# A rare case of thyroid cartilage chondroma: diagnostic challenges and the role of multimodal imaging and histopathology

**DOI:** 10.1093/bjrcr/uaag032

**Published:** 2026-08-03

**Authors:** Jitesh Arora, Zena A Ahmad

**Affiliations:** Department of Radiology, Queen’s Medical Centre, Nottingham University Hospitals NHS Trust, Nottingham, NG7 2UH, United Kingdom; Department of Radiology, Queen’s Medical Centre, Nottingham University Hospitals NHS Trust, Nottingham, NG7 2UH, United Kingdom

**Keywords:** chondroma, thyroid cartilage, laryngeal tumour, chondrosarcoma, multimodal imaging, neck mass, head and neck radiology

## Abstract

Chondromas are rare benign cartilaginous tumours, typically arising in the small bones of the hands and feet. Laryngeal chondromas are exceptionally uncommon and account for a very small proportion of head and neck neoplasms. We present a case of a 72-year-old male referred with a painless, slowly enlarging right-sided neck mass. Multimodal imaging, including ultrasound, CT, and MRI, revealed a well-circumscribed lesion with internal calcification arising from the thyroid cartilage. Differential diagnoses included chondroma and low-grade chondrosarcoma. Initial cytological assessment was inconclusive, prompting expert histopathological review, which confirmed a well-differentiated benign chondroid lesion consistent with a chondroma. The patient underwent successful surgical excision, and follow-up imaging over a 4-year period has shown no evidence of recurrence. This case highlights the diagnostic challenges in distinguishing chondromas from malignant cartilaginous tumours in atypical locations, and emphasises the importance of a multidisciplinary approach, combining imaging, histology, and specialist input to guide management.

## Case report

A 72-year-old male was referred by his general practitioner to the ear, nose, and throat (ENT) 2-week wait (2WW) clinic following a 4-week history of a progressively enlarging, painless neck lump. On clinical examination, a firm, well-defined, 3 cm mass was palpated on the right side of the neck, deep to the strap muscles in the submandibular region. The lesion moved with swallowing, raising suspicion of a lesion associated with the thyroid cartilage or adjacent structures. The patient denied associated symptoms such as dysphagia, hoarseness of voice, or dyspnoea, and there were no constitutional symptoms including weight loss, fever, or night sweats. He reported no recent infection or trauma. His past medical history was significant for prostate cancer, treated with radical prostatectomy a decade earlier, with no evidence of recurrence. There was no personal or family history of head and neck malignancy. He was a lifelong non-smoker, did not consume alcohol, and had an Eastern Cooperative Oncology Group (ECOG) performance status of 0, indicating full functional capacity.

**Figure 1:**
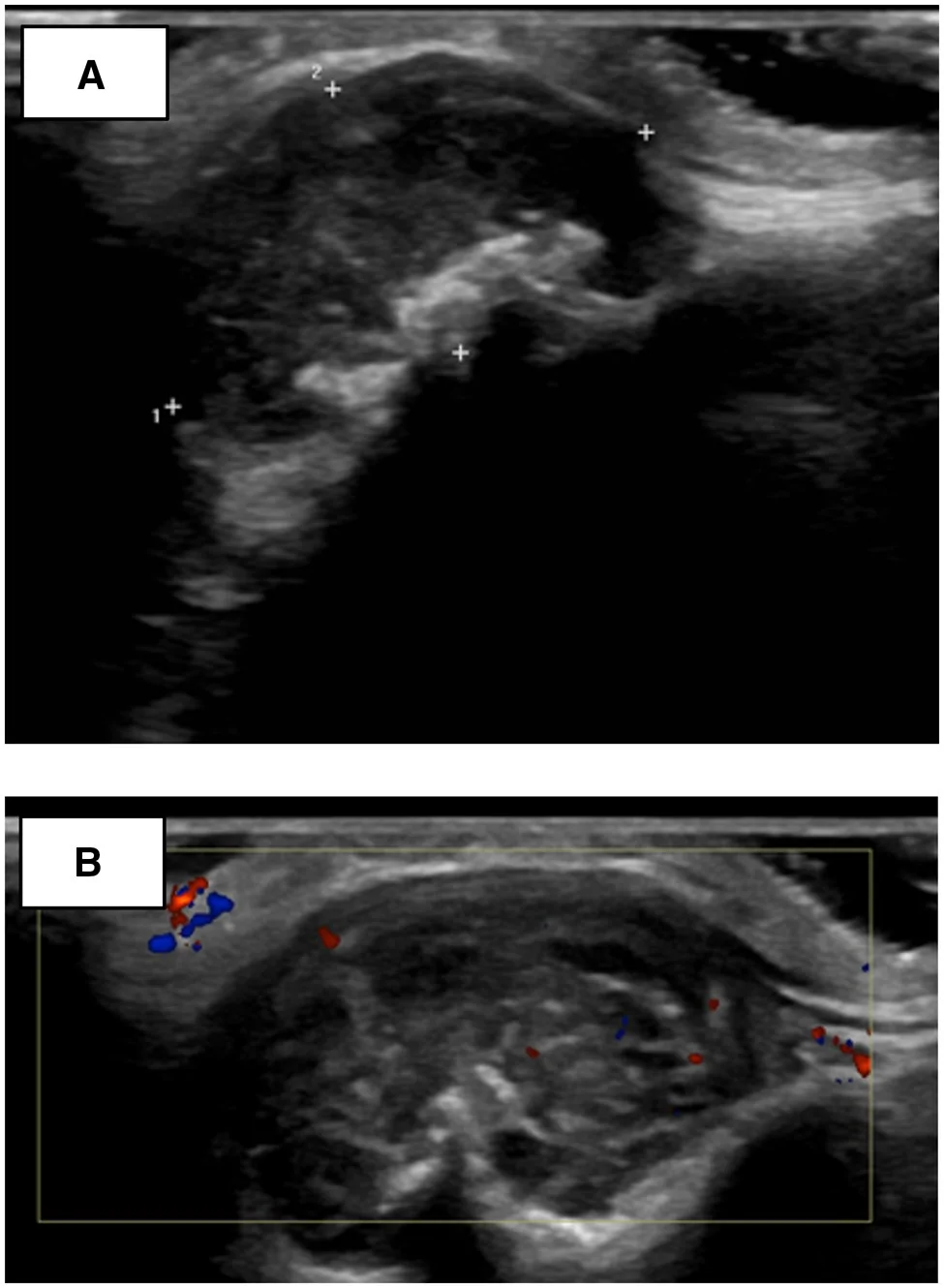
(A) High-resolution transverse ultrasound of the neck. Ultrasound demonstrates a 23 × 12 mm well-defined lesion with heterogeneous echogenicity at the level of the hyoid bone, containing internal calcific foci. The lesion appears closely related to the underlying cartilaginous structures and is separate from the thyroid gland. (B) Colour Doppler ultrasound of the neck. Colour Doppler ultrasound demonstrates minimal internal vascularity within the lesion, which measures approximately 23 × 12 mm and exhibits heterogeneous echogenicity with internal calcific foci at the level of the hyoid bone.

Routine laboratory investigations, including a full blood panel and thyroid function tests, were within normal limits. Initial imaging assessment involved a high-resolution transverse ultrasound of the neck at the right paramedian region (Figure 1A), which revealed a well-circumscribed, solid lesion measuring 23 × 12 mm in axial diameter. The mass exhibited heterogeneous echogenicity with internal calcifications and was suspected to originate from the hyoid bone. Colour Doppler imaging demonstrated minimal internal vascularity within the lesion (Figure 1B). Ultrasound-guided fine-needle aspiration (FNA) was performed during the same session. Based on these findings, the primary differential diagnoses included a benign chondroma versus low-grade chondrosarcoma. Less likely considerations included an infected thyroglossal duct cyst and metastatic disease secondary to the patient’s previous prostate cancer. No pathological cervical lymphadenopathy was identified on ultrasound.

**Figure 2:**
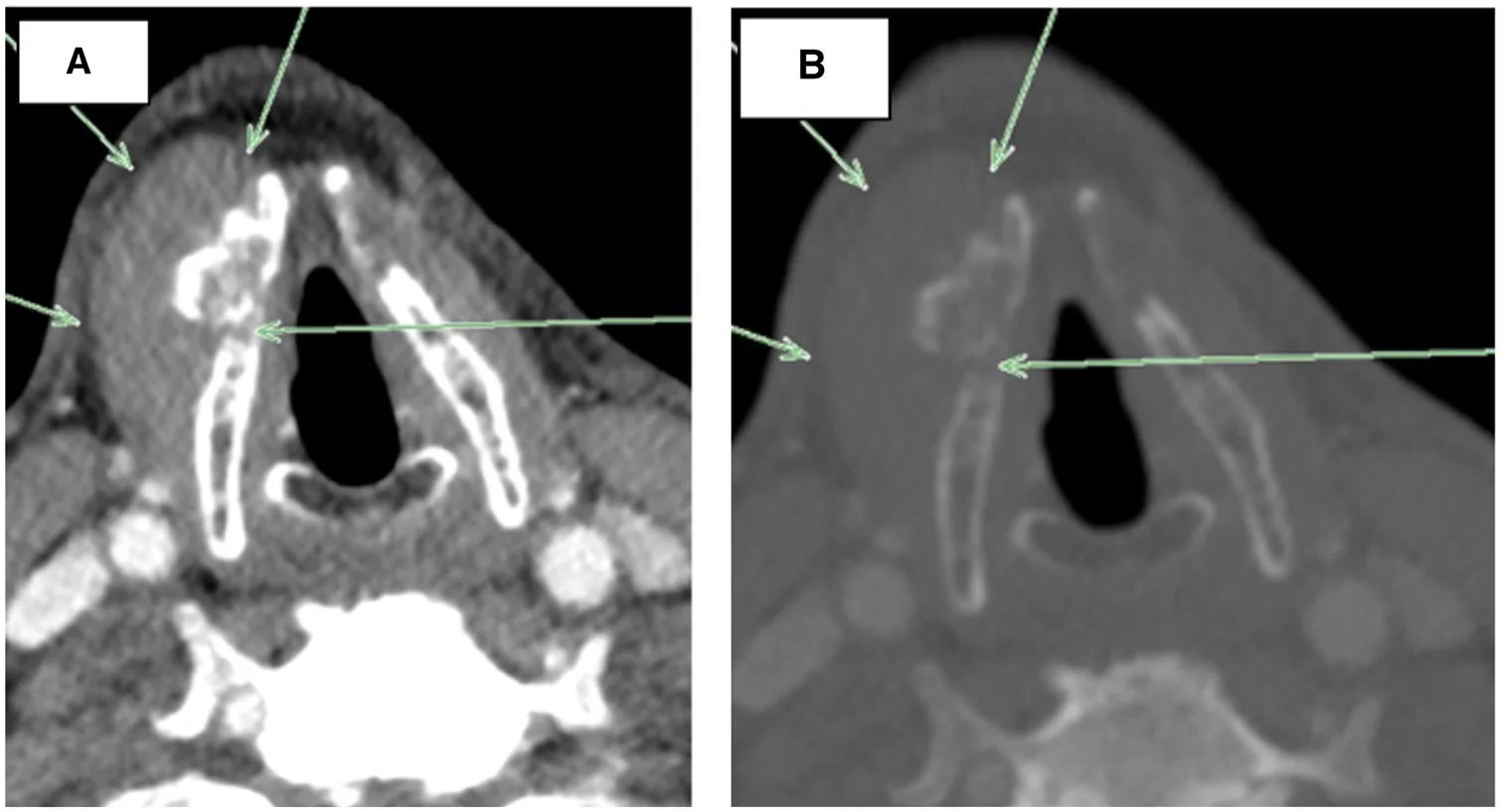
(A) Contrast-enhanced CT (soft tissue window) of the neck. Axial CT demonstrates an exophytic, well-defined, encapsulated soft tissue density mass arising from the anterosuperior aspect of the right thyroid cartilage lamina, containing foci of chondroid matrix mineralisation. There is no extension into the paraglottic space or laryngeal vestibule, and no evidence of aggressive soft tissue infiltration. (B) Contrast-enhanced CT (bone window) of the neck. Bone window CT further demonstrates the well-circumscribed exophytic lesion arising from the right thyroid cartilage lamina, with internal matrix calcification and no features of cortical destruction or aggressive bony erosion.

Further cross-sectional imaging was performed while awaiting histopathological results. Contrast-enhanced CT of the neck demonstrated a rounded, exophytic lesion arising from the anterosuperior aspect of the right thyroid cartilage (Figure 2A). The lesion produced focal expansion and remodelling of the adjacent thyroid cartilage lamina and contained internal chondroid matrix mineralisation. Importantly, there were no aggressive features such as permeative cartilage destruction, soft tissue invasion, or extension into the paraglottic space or laryngeal vestibule. Corresponding bone window images (Figure 2B) demonstrated the internal calcified matrix and confirmed the absence of aggressive osseous destruction. No cervical lymphadenopathy was identified.

**Figure 3:**
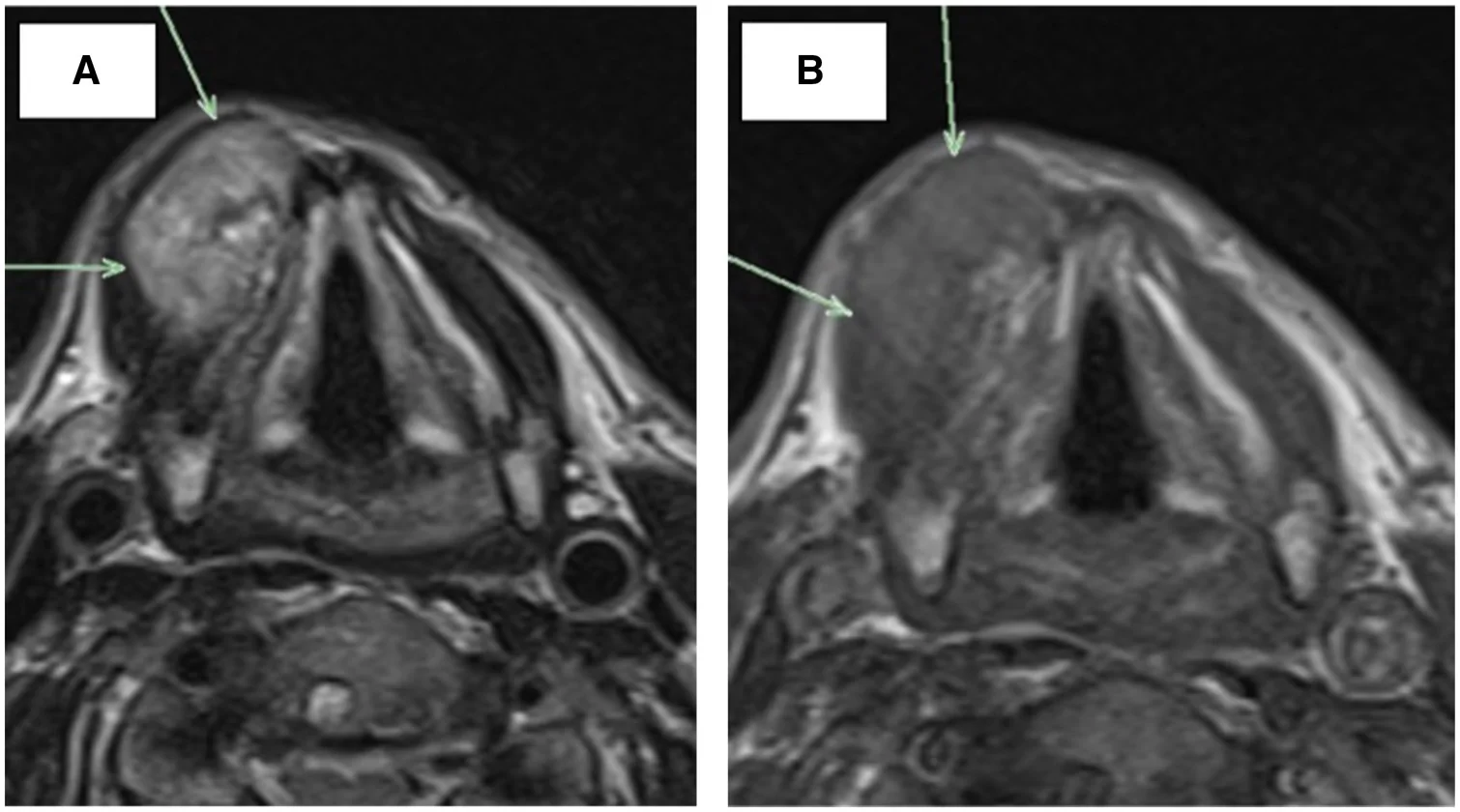
(A) Axial T2-weighted MRI of the neck. MRI demonstrates a 26 × 17 mm well-defined T2 hyperintense mass arising from the anterosuperior aspect of the right thyroid cartilage lamina. The lesion contains internal low-signal foci corresponding to calcifications and shows no extension into the paraglottic space or laryngeal vestibule. (B) Axial T1-weighted MRI of the neck. The lesion demonstrates intermediate signal intensity on T1-weighted imaging, with areas of internal higher signal intensity at its origin from the anterosuperior thyroid cartilage.

**Figure 4:**
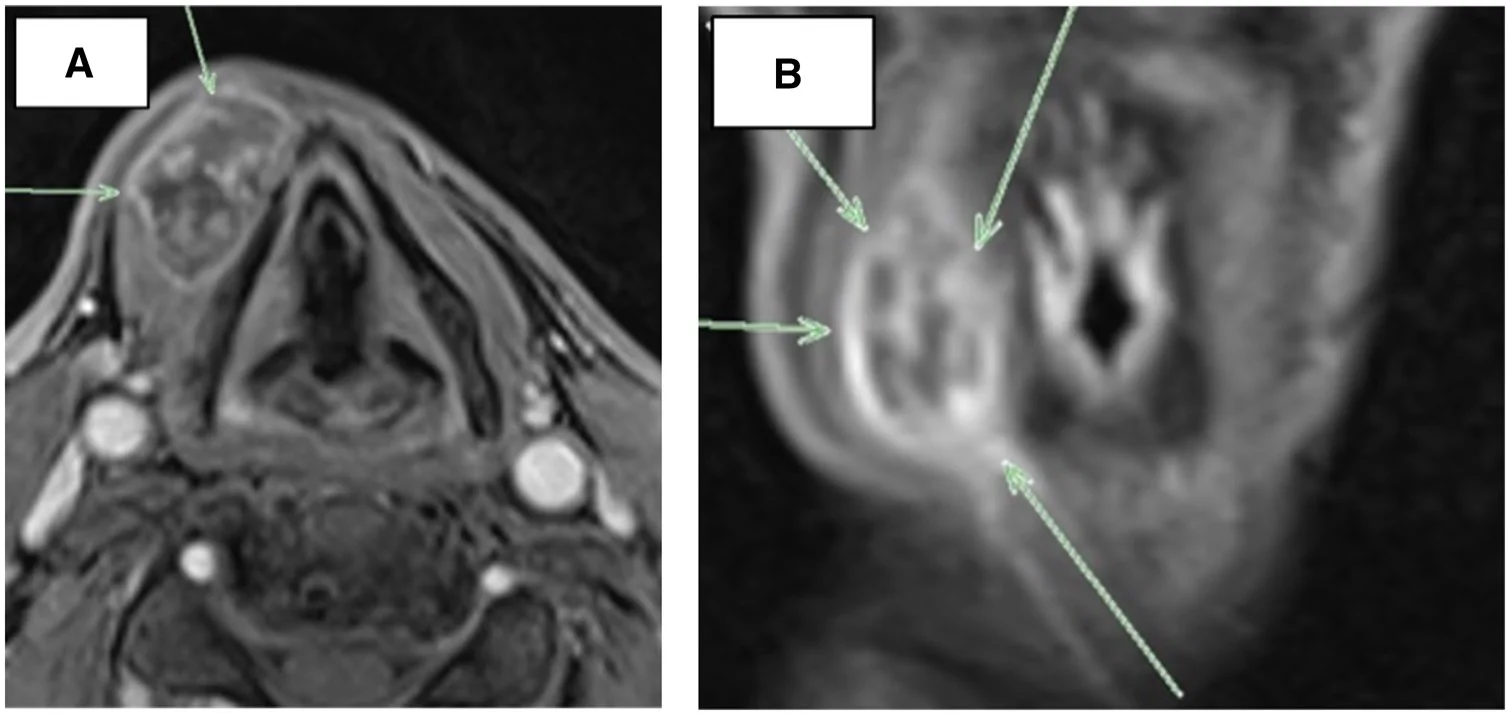
(A) Post-contrast axial T1-weighted MRI of the neck. Post-contrast T1-weighted imaging demonstrates heterogeneous enhancement of the mass lesion, which remains well defined and encapsulated without evidence of infiltration into adjacent soft tissues or surrounding structures. (B) Post-contrast coronal T1-weighted MRI of the neck. Coronal post-contrast imaging confirms the well-circumscribed enhancing lesion arising from the anterosuperior aspect of the right thyroid cartilage, with no invasion of the surrounding laryngeal structures.

Subsequent MRI of the neck with contrast provided further tissue characterisation. Axial T2-weighted images (Figure 3A) demonstrated a predominantly high signal intensity mass interspersed with focal areas of low signal, correlating with the calcific foci seen on CT. Axial T1-weighted images (Figure 3B) revealed isointense signal relative to adjacent strap musculature, again with punctate low-signal areas. Subtle signal alteration was noted within the adjacent right thyroid cartilage lamina at the site of tumour origin, reflecting focal cartilage involvement without features of cortical destruction or soft tissue infiltration. Post-contrast axial (Figure 4A) and coronal (Figure 4B) T1-weighted sequences showed heterogeneous enhancement, with a clearly defined, encapsulated peripheral rim and no evidence of perilesional soft tissue infiltration. No pathological cervical lymphadenopathy was identified on MRI.

The case was subsequently discussed at multidisciplinary team (MDT) meetings, where the lesion was described as an ovoid mass arising from the right lamina of the thyroid cartilage, exhibiting chondroid matrix mineralisation and a soft tissue component. While imaging findings were not overtly aggressive, chondrosarcoma could not be definitively excluded. Initial cytological analysis suggested an atypical cartilaginous tumour, prompting referral to a national specialist histopathology centre for bone and soft tissue tumours.

Fine-needle aspiration has well-recognised limitations in the evaluation of cartilaginous neoplasms, particularly when attempting to distinguish benign chondromas from low-grade chondrosarcomas. Cytological samples may provide limited tissue architecture and can demonstrate overlapping features between benign and malignant cartilaginous lesions. As a result, FNA findings are often insufficient for definitive diagnosis in isolation and must be interpreted in conjunction with imaging findings, clinical presentation, and definitive histopathological assessment following surgical excision. Expert review concluded that the lesion represented a benign or well-differentiated chondroid neoplasm, in keeping with a chondroma.

In light of the multidisciplinary consensus and specialist histopathological input, definitive surgical management was undertaken. The patient underwent open surgical excision of the lesion via a transcervical approach. Intraoperatively, a well-encapsulated mass arising from the anterosuperior aspect of the right thyroid cartilage lamina was identified. The lesion was excised together with the involved portion of the thyroid cartilage to achieve complete removal while preserving the structural integrity of the larynx. The procedure was completed without complication, and no intraoperative breach of the airway or significant haemorrhage was encountered.

Histopathological examination demonstrated a well-differentiated cartilaginous lesion composed of mature hyaline cartilage with low cellularity. There was no significant cytological atypia, binucleation, mitotic activity, necrosis, or myxoid matrix change. Importantly, the lesion demonstrated a well-circumscribed growth pattern without permeative or infiltrative extension into the surrounding tissues. Sections including adjacent thyroid cartilage confirmed that the lesion arose from the thyroid lamina without evidence of destructive infiltration or entrapment of pre-existing structures. Specialist review at a national bone and soft tissue pathology centre supported the diagnosis of a benign chondroma rather than low-grade chondrosarcoma.

**Figure 5:**
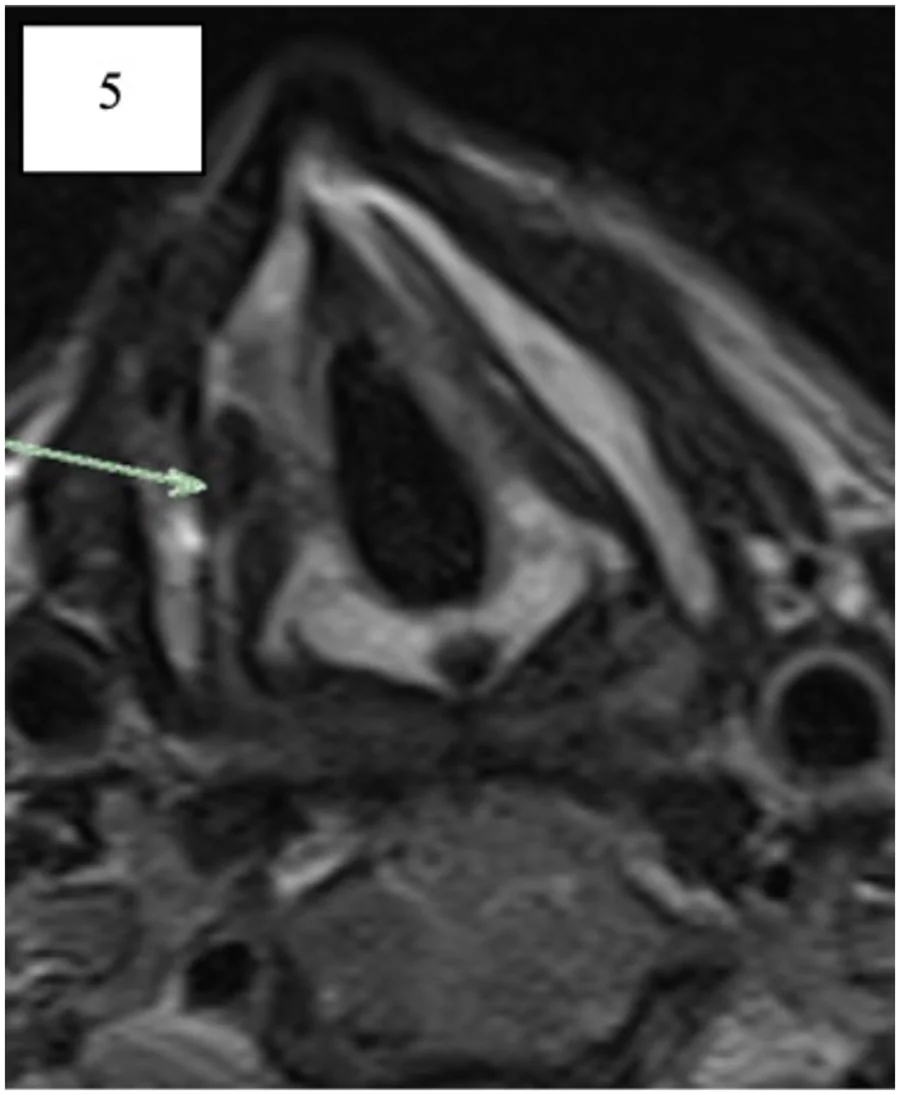
Follow-up axial T2-weighted MRI showing postoperative site. Follow-up imaging demonstrates low-signal intensity changes at the previous surgical site, consistent with postoperative fibrosis, with no radiological features suggestive of recurrent tumour.

Following surgical resection, the patient has undergone interval surveillance with annual contrast-enhanced MRI of the neck to assess for potential recurrence. Over a 4-year follow-up period, imaging appearances have remained stable with no evidence of disease progression. Most recently, axial T2-weighted sequences (Figure 5) demonstrated low signal intensity changes at the site of the previous surgery, consistent with postoperative fibrosis. The overall imaging pattern is in keeping with post-surgical scarring, with no radiological features suggestive of residual or recurrent tumour.

## Discussion

Chondromas are rare, benign mesenchymal tumours composed of mature hyaline cartilage, most commonly arising in the small bones of the hands and feet. Less frequently, they may occur within larger axial or appendicular skeletal sites, or in craniofacial regions such as the skull base and paranasal sinuses.^1^ These lesions are typically indolent and non-invasive, although rare instances of malignant transformation have been reported.^1^

Laryngeal chondromas are exceptionally uncommon, accounting for less than 1% of all laryngeal neoplasms and just 0.12% of head and neck tumours overall.^2^ When present, they most frequently originate from the cricoid cartilage, with less common involvement of the thyroid, arytenoid, or epiglottic cartilages.^2^ Chondromas arising from the thyroid cartilage, as in this case, are particularly rare and necessitate meticulous radiological and histopathological assessment to differentiate them from malignant mimics.

Multimodal imaging played a key role in the diagnostic work-up of this lesion. Ultrasound provided an initial assessment of the palpable neck mass and facilitated image-guided tissue sampling. CT was particularly valuable for demonstrating chondroid matrix mineralisation and defining the relationship of the lesion to the thyroid cartilage. MRI offered superior soft tissue contrast and allowed careful assessment of potential extension into the paraglottic space and adjacent laryngeal structures, which is valuable when assessing for features of low-grade chondrosarcoma. However, it is important to recognise that low-grade chondrosarcomas may also demonstrate relatively non-aggressive imaging appearances, with considerable overlap in radiological characteristics compared with benign chondromas. Consequently, imaging findings alone are insufficient for reliable differentiation, and definitive diagnosis frequently requires specialist histopathological evaluation.

The principal differential diagnosis in this context is chondrosarcoma. Although chondromas and chondrosarcomas represent distinct pathological entities, they can demonstrate significant overlap in clinical and imaging features, particularly in cases of low-grade chondrosarcoma. Hence, definitive diagnosis typically requires histopathological confirmation. A comparison of distinguishing features between chondromas and chondrosarcomas is summarised in Table 1^3^^,^^4^ encompassing clinical presentation, imaging characteristics across multiple modalities, and histological criteria.

**Table 1:** Comparative features of chondroma and chondrosarcoma.

	Chondroma	Chondrosarcoma
**Clinical features**	Slow-growing, painless mass; typically, an incidental finding.	Progressive enlargement with potential pain, dysphagia, hoarseness of voice, or airway compromise.
**Ultrasound findings**	Well-defined, hypoechoic lesion with posterior acoustic enhancement; no vascularity on Doppler.	Heterogeneous, irregular mass with internal vascularity; may show cortical destruction and adjacent soft tissue involvement with calcific foci.
**CT neck with contrast**	Well-circumscribed, low-attenuation lesion; usually shows calcification but lacks aggressive features.	Lobulated, destructive lesion with irregular margins, cortical disruption, lytic changes, and chondroid matrix calcifications (rings and arcs).
**MRI neck with contrast**	T2 hyperintense, T1 isointense, with scalloped margins and curvilinear translesional septal enhancement; smooth margins and no adjacent invasion.	T2 hyperintense with heterogeneous enhancement; may demonstrate ill-defined borders, cortical disruption, or local infiltration into adjacent soft tissues, although low-grade lesions can occasionally appear relatively well circumscribed.
**Histopathology**	Composed of mature hyaline cartilage; lacks mitotic activity, nuclear atypia, or necrosis.	Increased cellularity, nuclear atypia, mitotic activity, and myxoid matrix degeneration; may show an infiltrative growth pattern.

Given the considerable overlap in imaging features between benign and malignant cartilaginous tumours, this case highlights the critical role of a multidisciplinary approach incorporating clinical, radiological, and histopathological input. Accurate diagnosis is essential to avoid overtreatment or mismanagement, particularly in rare anatomical locations such as the thyroid cartilage.

## Learning points

- Chondromas are rare benign cartilaginous tumours that can present as slowly enlarging, painless neck masses, with exceptional cases arising from the thyroid cartilage.
- Distinguishing chondromas from low-grade chondrosarcomas is challenging due to overlapping clinical and imaging features, especially in atypical anatomical locations.
- A multimodal assessment including detailed imaging, cytology, and histopathological review is essential for accurate diagnosis and appropriate management.
- Multidisciplinary team input is vital in cases where diagnostic uncertainty exists, helping to guide the balance between surgical intervention and conservative management.
- Long-term imaging follow-up is important to monitor for recurrence or post-surgical changes, even in histologically benign cases.
